# Exenatide (a GLP-1 agonist) improves the antioxidative potential of in vitro cultured human monocytes/macrophages

**DOI:** 10.1007/s00210-015-1124-3

**Published:** 2015-05-19

**Authors:** Łukasz Bułdak, Krzysztof Łabuzek, Rafał Jakub Bułdak, Grzegorz Machnik, Aleksandra Bołdys, Bogusław Okopień

**Affiliations:** Department of Internal Medicine and Clinical Pharmacology, School of Medicine in Katowice, Medical University of Silesia, Medykow 18, 40-752 Katowice, Poland; Department of Physiology, School of Medicine with the Division of Dentistry in Zabrze, Medical University of Silesia, Jordana 19, 41-808 Zabrze, Poland

**Keywords:** Exenatide, GLP-1, LPS, Superoxide dismutase, Glutathione peroxidase, Catalase

## Abstract

Macrophages are dominant cells in the pathogenesis of atherosclerosis. They are also a major source of reactive oxygen species (ROS). Oxidative stress, which is particularly high in subjects with diabetes, is responsible for accelerated atherosclerosis. Novel antidiabetic drugs (e.g., glucagon-like peptide-1 (GLP-1) agonists) were shown to reduce ROS level. Therefore, we conceived a study to evaluate the influence of exenatide, a GLP-1 agonist, on redox status in human monocytes/macrophages cultured in vitro, which may explain the beneficial effects of incretin-based antidiabetic treatment. Human macrophages obtained from 10 healthy volunteers were in vitro subjected to the treatment with GLP-1 agonist (exenatide) in the presence of lipopolysaccharide (LPS), antagonist of GLP-1 receptors (exendin 9-39), or protein kinase A inhibitor (H89). Afterwards, reactive oxygen species, malondialdehyde level, NADPH oxidase, and antioxidative enzymes [superoxide dismutase (SOD), glutathione peroxidase (GSH-Px), and catalase] expression was evaluated. Finally, we estimated the activity of the abovementioned enzymes in the presence of H89. According to our findings, exenatide reduced ROS and malondialdyhyde (MDA) level by decreasing the expression of ROS-generating NADPH oxidase and by increasing the expression and activities of SOD and GSH-Px. We also showed that this effect was significantly inhibited by exendin 9-39 (a GLP-1 antagonist) and blocked by H89. Exenatide improved the antioxidative potential and reduced oxidative stress in cultured human monocytes/macrophages, and this finding may be responsible for the pleiotropic effects of incretin-based therapies. This effect relied on the stimulation of GLP-1 receptor.

## Introduction

Since several years, novel groups of antidiabetic drugs emerge (SGLT-2 inhibitors, DPP-IV inhibitors, and glucagon-like peptide-1 (GLP-1) agonists). The most promising results are connected with the incretin-based treatment, especially GLP-1 agonists. These drugs not only reduce blood glucose but also decrease body weight and insulin resistance. Additionally, significant reductions in reactive oxygen species (ROS) level, the expression of proinflammatory cytokines, and the shift toward an alternative, antiinflammatory phenotype were noted on GLP-1 agonist-based therapies (Chaudhuri et al. 2012).

The substantial portion of deleterious effects of diabetes results from oxidative stress. It was shown that ROS damage beta islet cells and reduce the excretion of insulin (Mukai et al. 2011). Additionally, ROS impair insulin sensitivity in peripheral tissues and are directly or indirectly associated with the multistage process of atherogenesis. Macrophages are key sources of oxidative stress. They are immune cells involved in the innate defensive mechanisms against pathogens. Nevertheless, their role in the pathogenesis of atherosclerotic plaques and diabetes is substantial. Nowadays, at least two distinct populations of macrophages are recognized. Classically activated macrophages are associated with inflammatory response, a high level of ROS generation, and an elevated level of proinflammatory cytokine secretion. These cells are formed due to lipopolysaccharide (LPS) stimulation and also during low-grade chronic inflammation that is seen in atherosclerotic plaques, where they transform into foam cells (Kaplan et al. 2012). The second population, called alternatively activated macrophages, is responsible for healing and putting down inflammatory response. In vivo, these cells are also formed during parasitic infestation, but in an in vitro setting, IL-4 is used (Van Dyken and Locksley 2013). The modification of the function of macrophages seems to be a promising target for the treatment of diseases resulting from atherosclerosis. Data regarding the contribution of macrophages in the progression of cardiovascular diseases have been available for around 20 years (Stein et al. 1992). However, only recently have we shown that alternative activation may be obtained using various drugs that have pleiotropic effects (i.e., effects stretching beyond the main mechanism of action) (Labuzek et al. 2014). Alternatively activated macrophages are characterized by the increased expression of antioxidative enzymes (superoxide dismutase [SOD], glutathione peroxidase [GSH-Px], catalase [CAT]) (Nagashima et al. 2011). These antioxidant enzyme systems belong to regulatory mechanisms that protect against oxidative stress. Under oxidative stress, antioxidant enzymes rapidly eliminate ROS (Park and Oh 2011). The imbalance between the oxidative processes and antioxidant system activity may lead to the deterioration of atherosclerosis. Recent studies have shown that GLP-1 agonists reduce the extent of oxidative stress by reducing ROS levels in H9c2 myocytes and in human monocytes (Chang et al. 2014; Ishibashi et al. 2011) or by decreasing NADPH oxidase activity (Ojima et al. 2012). However, the reason for the observed influence of GLP-1 agonism on redox status is unknown.

According to the abovementioned data, we conceived a study on the effects of exenatide (a GLP-1 agonist) on the redox potential of human monocytes/macrophages. Therefore, we assessed the synthesis of ROS in the cultured cells treated with LPS and exenatide. Then, we estimated the malondialdyhyde (MDA) level in monocyte/macrophage cultures as a marker of oxidative burden. Afterwards, we assessed the expression of an oxidative enzyme (p22 NADPH oxidase) and antioxidative enzymes (GSH-Px, CAT, SOD) and activities of antioxidative enzymes (GSH-Px, CAT, SOD). Finally, an experiment estimating effects of H89 on antioxidative enzymes’ activities was performed.

## Materials and methods

### Cell culture

Peripheral blood mononuclear cells were separated by histopaque density gradient centrifugation from the human group including 10 healthy nonsmoking volunteers aged 18–40 years, 5 women and 5 men not taking any drugs, using a previously described methods (Okopien et al. 2005). Then, monocytes were isolated from peripheral blood mononuclear cells by negative immunomagnetic separation using Pan-T and Pan-B Dynabeads (Dynal, Oslo, Norway). This procedure enabled us to isolate inactive monocytes without artificial and uncontrolled stimulation. The isolated cells were labeled with a monoclonal antibody (Daco, Glostrup, Denmark) against the monocyte-specific positive antigen CD14+. The procedure gave 92 % of CD14+ positive cells in the isolated fraction. Monocytes were suspended in RPMI 1640 medium supplemented with 10 % FCS (low endotoxin), 2 mM glutamine, 100 U/ml penicillin, 100 mg/ml streptomycin, and 10 mg/ml fungizone (Gibco, Grand Island, NY, USA). The cells were counted using an automated cell counter TC-20 (Bio-Rad, Hercules, CA, USA). A constant number of cells (10^6^ monocytes per well) was placed in a plastic 24-well plate (Becton-Dickinson, Franklin Lakes, NJ, USA) and left intact for 2 h to allow them to adhere to the bottom. Then, the medium was changed, and cultures were incubated for 72 h (with single medium exchange after first 24 h). Incubations were performed in triplicate at 37 °C in a humidified atmosphere containing 5 % CO_2_ in the air. The conversion of monocytes into macrophages was confirmed using antibodies against EMR1 (Sigma-Aldrich, Poznan, Poland). After a 72-h incubation, the supernatant was carefully removed and replaced with medium supplemented with selected reagents (all supplied by Sigma-Aldrich, Poznan, Poland) dissolved in culture medium: inducer of inflammatory state—LPS (0.5 μg/ml), GLP-1 agonist—exenatide (10 nM), and GLP-1 antagonist—exendin 9-39 (50 nM), protein kinase A (PKA) inhibitor—H89 (10 μM).

### Viability tests

The viability of cells was estimated in two separate tests. The first was based on 0.4 % trypan blue exclusion test according to manufacturer’s guidelines. Briefly, aliquots (10 μl) of cultured cells suspended in the medium were mixed with 10 μl of 0.4 % trypan blue. After the incubation, cells were loaded on a slide and the viability was assessed in the automated cell counter TC-20 (Bio-Rad, Hercules, CA, USA). The second method relied on tetrazolium salt (MTT) conversion. MTT (final concentration 2.5 mg/ml) was added to the medium 3 h before the scheduled end of the experiment, and then, the cultures were incubated at 37 °C, 5 % CO_2_/95 % air in proper conditions. At the end of the experiment, after being washed twice with PBS, monocytes were lysed in 100 μl of dimethyl sulfoxide, which enabled the release of the blue reaction product—formazan (room temperature, 10 min in the dark). The lysate (200 μl) was transferred to a 96-well plate (Falcon 353072, Becton-Dickinson, Franklin Lakes, NJ, USA). Absorbance at the wavelength of 570 nm was read using a microplate reader (Dynex Technologies, VA, USA) in three measurements in 10 independent experiments. The results were expressed as a percentage of the control (100 %).

### ROS measurement

Monocytes incubated in 24-well tissue culture plates (10^6^ cells per well) were treated with studied compounds (37 °C, 5 % CO_2_/95 % air). After 24 h, the cells were removed from the wells by trypsin, collected, and resuspended in DMEM containing 1 mg/ml nitro blue tetrazolium (NBT) (Sigma–Aldrich, Poznan, Poland). Next, monocytes were lysed using distilled water and brief sonication. Aliquots of the samples were added to 96-well plates, and NBT reduction was measured by absorbance at 550 nm in triplicate using a microplate reader in 10 independent experiments. The results were expressed as a percentage of the control (100 %).

### RT-QPCR

The relative gene expression of GSH-Px, CAT, SOD, and p22 NADPH oxidase was measured by a two-step, reverse transcription quantitative polymerase chain reaction (RT-QPCR) normalized against the expression level of the glyceraldehyde 3-phosphate dehydrogenase (GAPDH) gene after 8 h of incubation. Total RNA was extracted from cells using TriPure Isolation Reagent (Roche Diagnostics GmbH, Mannheim, Germany) according to a standard protocol. Concentrations, as well as quality of RNA extracts, were estimated by spectrophotometry (at 260 and 280 nm, BioPhotometer, Eppendorf, Germany). An amount of 1 μg of total RNA of each sample was reverse transcribed using the MMLV Reverse Transcriptase First-Strand complementary DNA (cDNA) Synthesis Kit (Epicentre Technologies, Madison, USA). A final RT reaction volume of 20 μl was diluted five times in order to avoid possible reaction inhibition by high levels of cDNA. The quantitative polymerase chain reaction was conducted using Brilliant II SYBR Green QRT-PCR 2 master mix (Agilent Technologies, Inc., Santa Clara, CA, USA). Reaction mixes containing one master mix, 300 nM of each (forward and reverse) primer complementary to GSH-Px, CAT, SOD, p22, or GAPDH, respectively, 4 μl of cDNA mixture (i.e., an equivalent of 40 ng of total RNA) in a total volume of 25 μl were analyzed with a LightCycler 480 Real-Time PCR System using a standard two-step thermal profile for QPCR (95 °C for 2 min, then 40 cycles of 95 °C for 15 s and 60 °C for 30 s. After amplification, a melting curve was plotted for each sample in order to confirm the specificity of the reaction. Primers used for quantitative analysis were designed using eprimer3 software (EMBOSS platform) according to the appropriate gene received from the GenBank database. The GAPDH primer pair was previously published and was retrieved from the primer database. Eprimer3, as well as Primersearch and Water software, was used for primer designing and comparisons and are freely available on the server of the Pasteur Institute (Paris, France http://mobyle.pasteur.fr) as a part of the European Molecular Biology Open Software Suite [EMBOSS] (Rice et al. 2000). Additionally, randomly chosen samples derived from QPCR assays were loaded onto 1.3 % agarose gels (Agagel Mini, Biometra, Goettingen, Germany) in order to confirm the specificity of amplification. No other bands than expected for a particular primer pair were visible.

### Protein extraction and Western blotting

Total protein concentrations in samples were determined spectrophotometrically. Bovine serum albumin preparations (Fermentas, Vilnius, Lithuania) were used for calculation of the standard curve. Equal amounts of total protein (50 μg) mixed 1:1 with 2× sample buffer (25 % glycerol, 2 % sodium dodecyl sulfate, 0.02 % bromophenol blue) were boiled for 6 min and loaded onto a 10 % SDS-polyacrylamide. Electrophoresis was continued at 180 V (at constant voltage) until bromophenol blue dye reached the end of the gel. After electrophoresis, the stacking gels were removed and resolved, and sample gels were directly subjected to Western blotting. After separation in polyacrylamide gels, the aliquots were transferred to polyvinylidene fluoride membranes (Pall Poland Ltd., Warszawa, Poland). Nonspecific antibody binding was inhibited by incubation in 20 mM Tris-buffered saline (pH 7.5) with 0.1 % Tween 20 (TBST) containing 5 % nonfat dried milk for 1 h at room temperature (RT). Polyclonal antibodies against GLP-1 receptor, GSH-Px, CAT, SOD, and p22 were obtained from Santa Cruz Biotechnology (Santa Cruz, CA, USA). The antibodies were diluted in TBST containing 5 % skim milk. The membranes were incubated with the antibodies overnight at 4 °C, washed with TBST, incubated at RT for 60 min with the appropriate alkaline phosphatase-conjugated secondary antibodies diluted 1:1000 (Bio-Rad Laboratories Inc. Hercules, CA, USA), and washed twice with TBST for 5 min and once with TBS for 5 min [20 mM Tris-buffered saline (pH 7.8)]. In each assay, the colored precipitates were developed directly on the membrane using AP-chromogenic substrates (Bio-Rad Laboratories, Hercules, CA, USA). All of the membranes were photocopied and subjected to further analysis. The molecular weights of GSH-Px, CAT, SOD, and p22 were confirmed according to their protein markers (PageRuler Unstained Protein Ladder, Fermentas, Vilnius, Lithuania). To control for the amounts of cytosolic proteins loaded in each lane, alpha-actin was detected in parallel using a 1:5000 dilution of anti-alpha-actin antibodies (Abcam Inc. Cambridge, MA, USA). The integrated optical density (IOD) of signals was semi-quantified using Image-Pro Plus software and is expressed as the ratio of the IOD for the tested proteins to the IOD for alpha-actin. The experiment was repeated three times, and the relative density values were subjected to statistical analysis.

### MDA assay

This product of lipid peroxidation was determined by the thiobarbituric acid (TBA) reaction. The reaction buffer (1.5 ml) contained 50 μg of protein from each sample and 1.4 ml of 0.2 M Tris–0.16 M KCl (pH 7.4) that had been incubated at 37 °C for 30 min, after which 1.5 ml of TBA reagent was added, and the mixture was then heated in a boiling water bath for 10 min. After cooling with ice, 3 ml of pyridine:*n*-butanol (3:1, *v*/*v*) and 1 ml of 1 M NaOH were added and mixed by shaking. The absorbance was read at 548 nm. The blank control contained the same reaction mixture but without cells. The level of malondialdehyde (MDA) was expressed as millimoles MDA per milligram of protein (mmol MDA/mg p).

### Enzyme activity assays

#### SOD activity assay

Cultured macrophages/monocytes after treatment with study drugs were cultured for 24 h. Afterwards, SOD activity was evaluated using a method described by Bułdak et al. (2013). Cells from wells of 24-well plastic dishes (10^6^/well) were harvested, lysed, sonicated for 10 s, and centrifuged (2000 rpm, 5 min). Protein concentration was measured using the Bio-Rad protein reagent (Bio-Rad, Warsaw, PL). The same volume from each sample containing 30 μg protein was mixed with 0.8 ml of 1× TDB (pH 7.4), 40 μl of 7.5 mM NADPH, and 25 μl of 100 mM EDTA:MnCl_2_ (2:1, POCh, PL). The reaction was initiated by addition of 0.1 ml of 10 mM mercaptoethanol (POCh, PL). The decrease in absorbance at 340 nm was recorded over 20 min at room temperature. The control consisted of a reaction mixture in which the study compound was replaced by an equal volume of the cell lysis buffer. The enzymatic activity of SOD was expressed in nitric units (NU) per milligram of protein (NU/mg p). One NU represents 50 % inhibition by SOD of nitrosol ion formation under these conditions. Three repeats of each test were performed in 10 independent culture experiments. In experiments requiring PKA inhibition, H89 was added to culture medium 30 min prior to the rest of studied compounds.

#### GSH-Px activity assay

The method of Paglia and Valentine was used with minor modifications. Briefly, monocytes/macrophages were harvested from culture wells. After centrifugation, the cell pellet was mixed with the cell lysis buffer and then sonicated for 10 s. Protein concentration was measured using the Bio-Rad protein reagent. The same volume from each sample containing 30 μg of protein was mixed with 2.68 ml of 0.05 M phosphate buffer (pH 7.0) containing 0.005 M EDTA. The following solutions were then added: 0.1 ml of 0.0084 M NADPH, 0.01 ml GSSG-R, 0.01 ml of 1.125 M NaNO_3_, and 0.1 ml of 0.15 M GSH. The enzymatic reaction was initiated by addition of 0.1 ml of 0.0022 M H_2_O_2_. The conversion of NADPH to NADP was followed by a continuous recording of the changes in absorbency at 340 nm between 2 and 4 min after initiation of the reaction. Control determinations were made by simultaneous assay with replacement of the sample by an equal volume of the cell lysis buffer. The activity of GSH-Px was determined as the number of micromoles of NADPH used for the regeneration of GSH within 1 min, recalculated per milligram of protein (IU/mg p). In experiments requiring PKA inhibition, H89 was added to culture medium 30 min prior to the rest of studied compounds.

#### CAT activity

Catalase activity was measured spectrophotometrically. Direct disappearance of 10 mM hydrogen peroxide in 50 mM potassium phosphate buffer (pH 7.0) containing 1 mM EDTA was measured at 240 nm over 30 s on a Beckman DU-70 spectrophotometer. Enzyme activity was calculated based on the molar extinction coefficient of hydrogen peroxide at 240 nm (*e* = 39.4 M^−1^ cm^−1^) and reported as micromole hydrogen peroxide decomposed per minute, recalculated per milligram of protein (kIU/mg p). In experiments requiring PKA inhibition, H89 was added to culture medium 30 min prior to the rest of studied compounds.

### Institutional review board

The ethical committee of the Medical University of Silesia accepted the study protocol.

### Statistical analysis

Results are expressed as the mean ± standard deviation (SD). The normality of distribution was checked by means of Shapiro–Wilk’s test. The statistical analysis of the data was performed using one-way ANOVA followed by the post hoc Tukey honestly significant difference test or Kruskal–Wallis test with Mann–Whitney tests according to parameter distribution. The Bonferroni adjustment was applied for multiple comparisons. Differences were considered significant for *p* < 0.05. Statistical analysis was performed using a SPSS statistical software package (SPSS 16.0 for Windows).

## Results

### GLP-1 receptors and viability (Fig. 1)

**Fig. 1:**
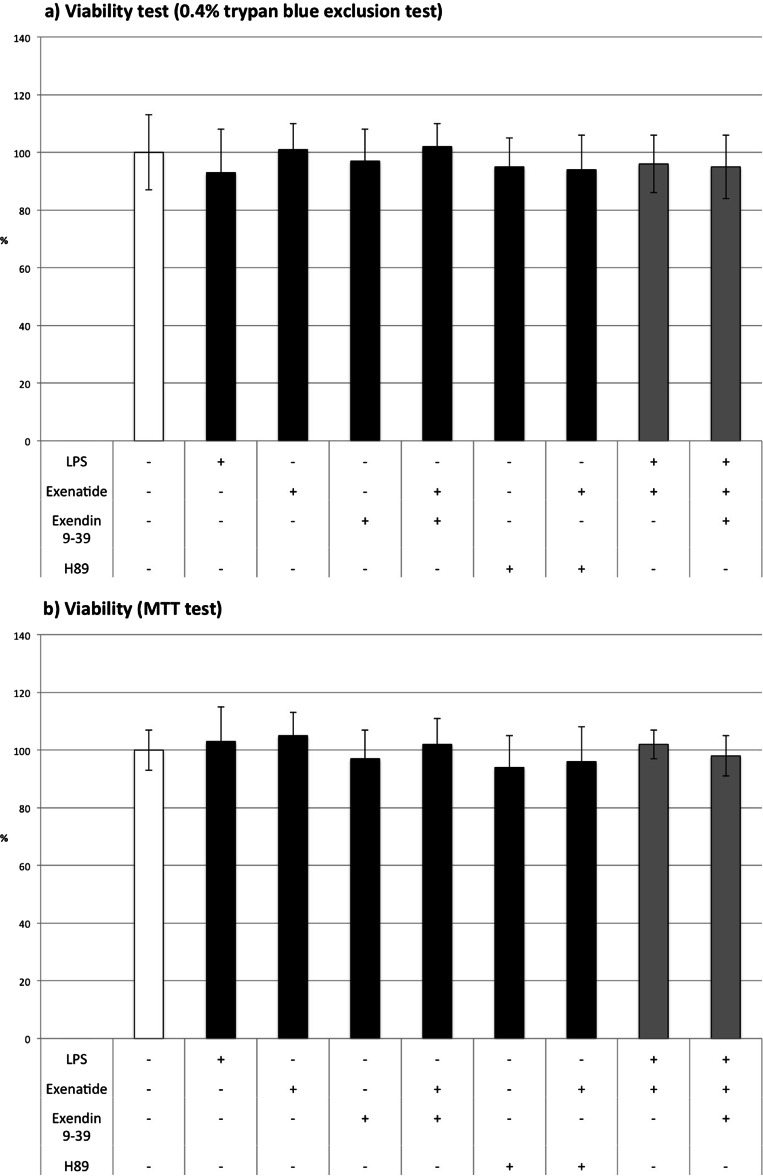
The viability of cultured monocytes/macrophages assessed by the 0.4 % trypan blue exclusion test and MTT

None of the reagents affected the viability of cultured cells compared to controls in both employed tests in the study. According to our Western blot analysis, we confirmed the expression of GLP-1 receptors on macrophages using a GLP-1-specific primary antibody, which showed a specific band at 53 kDa.

### ROS (Fig. 2a)

**Fig. 2:**
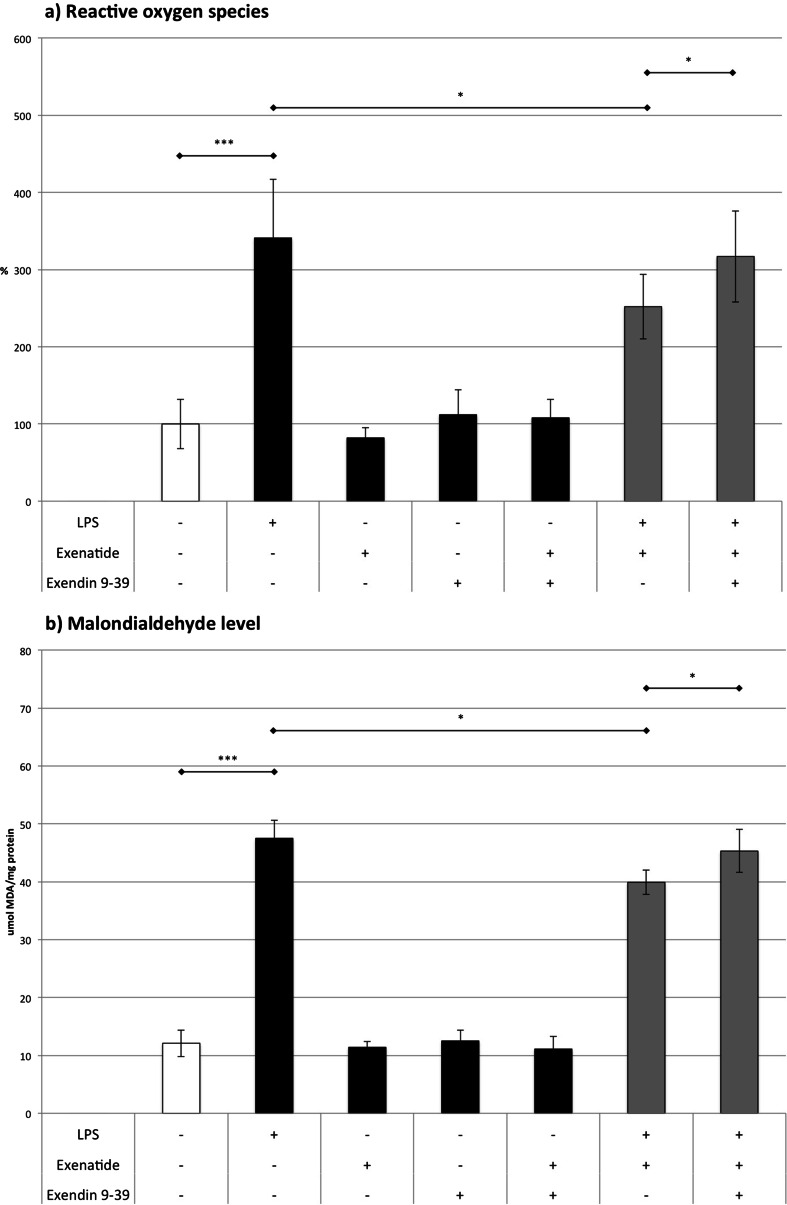
Effects of LPS, exenatide, and exendin 9-39 on **a** ROS level in the cultured monocytes/macrophages and **b** nitrite concentration in the cultured medium derived from macrophages’ cultures. *p < 0.05, **p < 0.01, ***p < 0.001

Results show massive (3.4-fold) increase in ROS generation that followed the exposure of monocytes/macrophages to LPS compared to controls. Contrary to these findings, exenatide did not affect the ROS level. Conversely, exenatide reduced the excess of ROS in cells treated with LPS by 26 %. The administration of GLP-1 receptor antagonist (exendin 9-39) abolished the effects of exenatide in LPS-treated cultures resulting in an ROS level that was similar to cells only subjected to LPS.

### MDA (Fig. 2b)

LPS treatment resulted in a nearly 4-fold increase in MDA level compared to untreated cells. Exenatide did not change the MDA level. However, it modestly reduced (by 16 %) the increase in MDA induced by LPS. Exendin 9-39 completely blocked the influence of exenatide on MDA in LPS-stimulated cells compared to cells only treated with LPS.

### NADPH oxidase (Fig. 3)

**Fig. 3:**
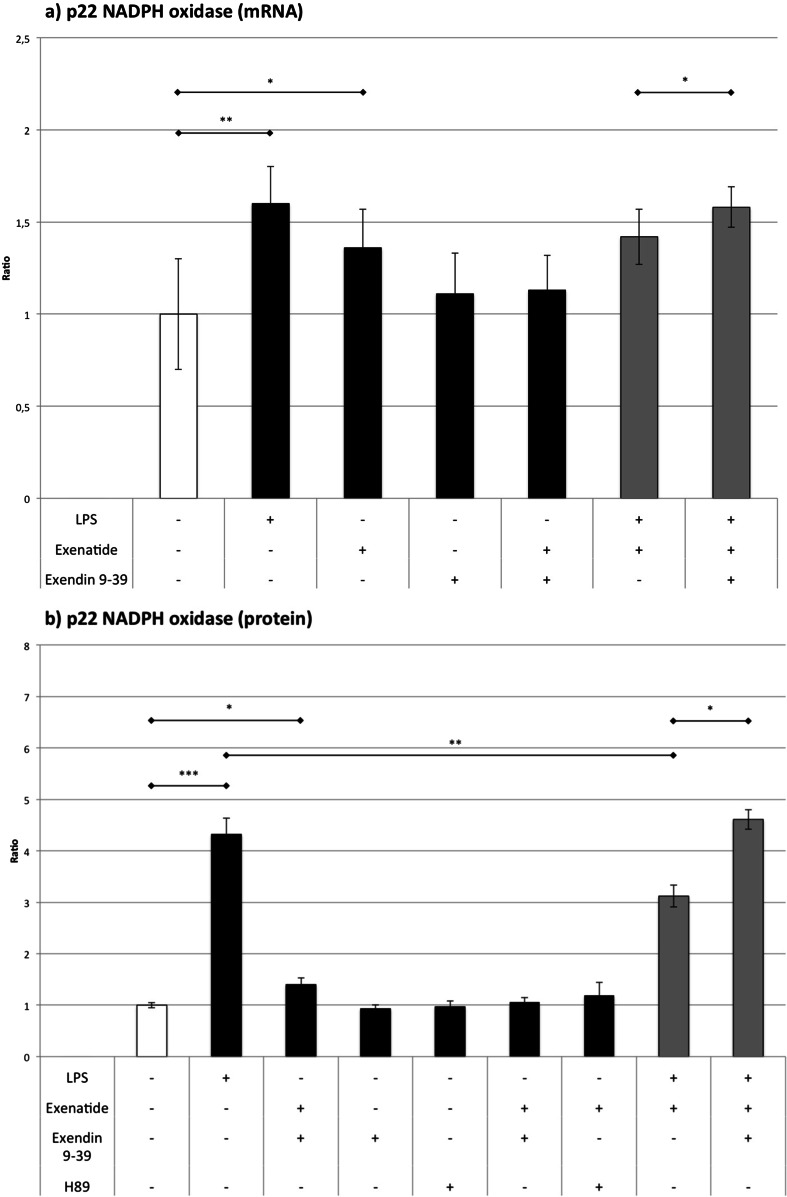
The influence of LPS, exenatide, exendin 9-39, and H89 (for Western blot only) on the expression of p22 NADPH oxidase assessed by **a** RT-QPCR and **b** Western blot analysis. *p < 0.05, **p < 0.01, ***p < 0.001

LPS elevated the messenger RNA (mRNA) and protein level of p22 by 60 and 432 %, respectively. Exenatide also significantly elevated the mRNA by 36 % and protein by 40 % compared to controls. However, exenatide reduced the impact of LPS on the protein expression of p22 by 28 % without any significant influence on the mRNA level for p22. The addition of exendin 9-39 abolished the effect of exenatide in macrophages treated with LPS.

### SOD (Fig. 4)

**Fig. 4:**
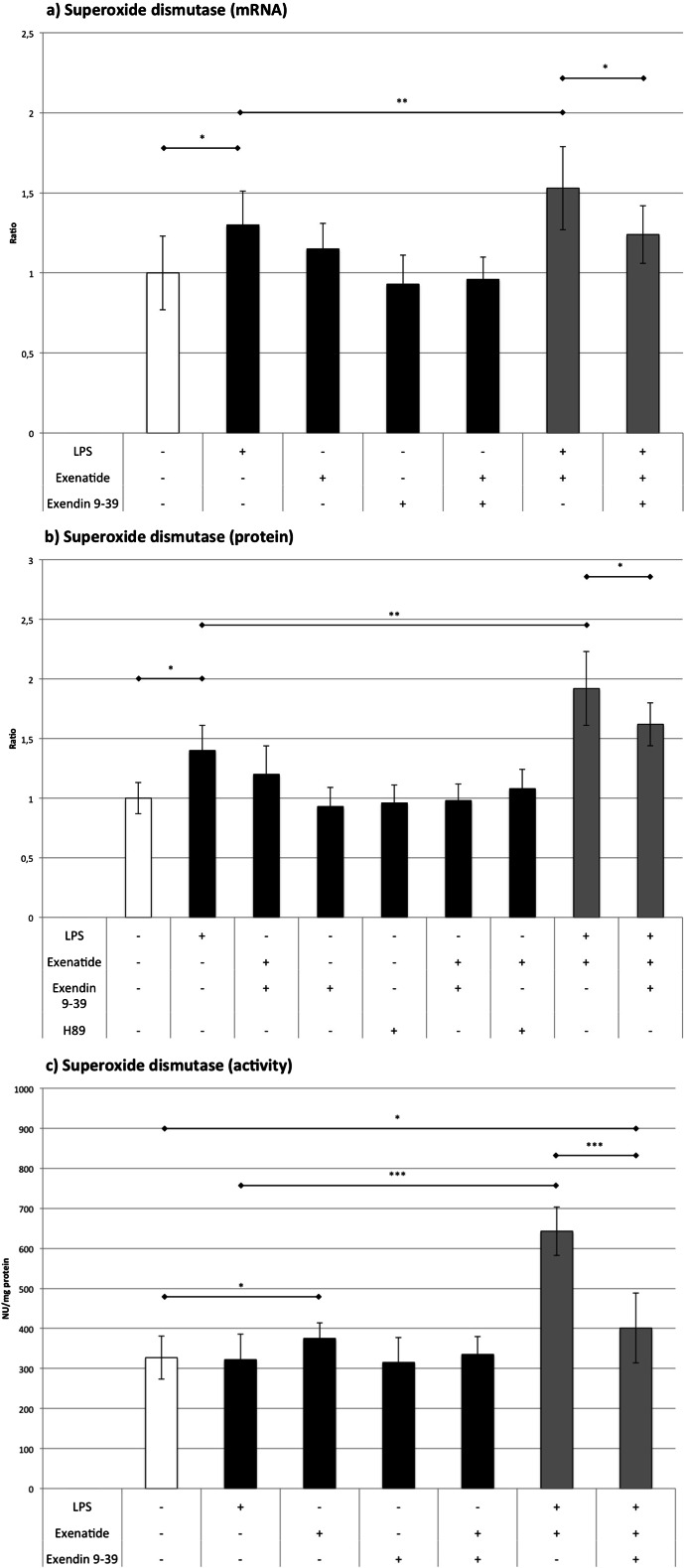
The influence of LPS, exenatide, exendin 9-39, and H89 (for Western blot only) on the expression of superoxide dismutase assessed by **a** RT-QPCR and **b** Western blot analysis and **c** on the activity of the superoxide dismutase. *p < 0.05, **p < 0.01, ***p < 0.001

LPS increased the quantity of mRNA and protein of SOD by 30 and 41 % without affecting its activity. This discrepancy between the expression level and lack of enzymatic activity may result from the decrease in antioxidative potential during LPS stimulation, which is antagonized in cells by an increase in the production of enzymes. Exenatide alone showed only a trend toward an increase in the expression of mRNA and protein but modestly increased the activity of SOD by 15 %. The most interesting results were observed in macrophages treated with exenatide plus LPS. Compared to cells treated only with LPS, a minute increase in mRNA level was seen (by 18 %), accompanied by a strong increase in protein synthesis (by 36 %), resulting in doubling the activity of SOD. Exendin 9-39 prevented a rise in mRNA and protein level in macrophages treated with LPS and exenatide compared to cells exposed only to LPS. SOD activity was reduced by exendin 9-39 (by 38 %) in cells subjected to LPS plus exenatide but remained elevated by 25 % compared to cultures treated with LPS. The incomplete inhibition of the activity by exendin 9-39 may result from the insufficient blockade of GLP-1 receptors (i.e., competition between agonist and antagonist).

### GSH-Px (Fig. 5)

**Fig. 5:**
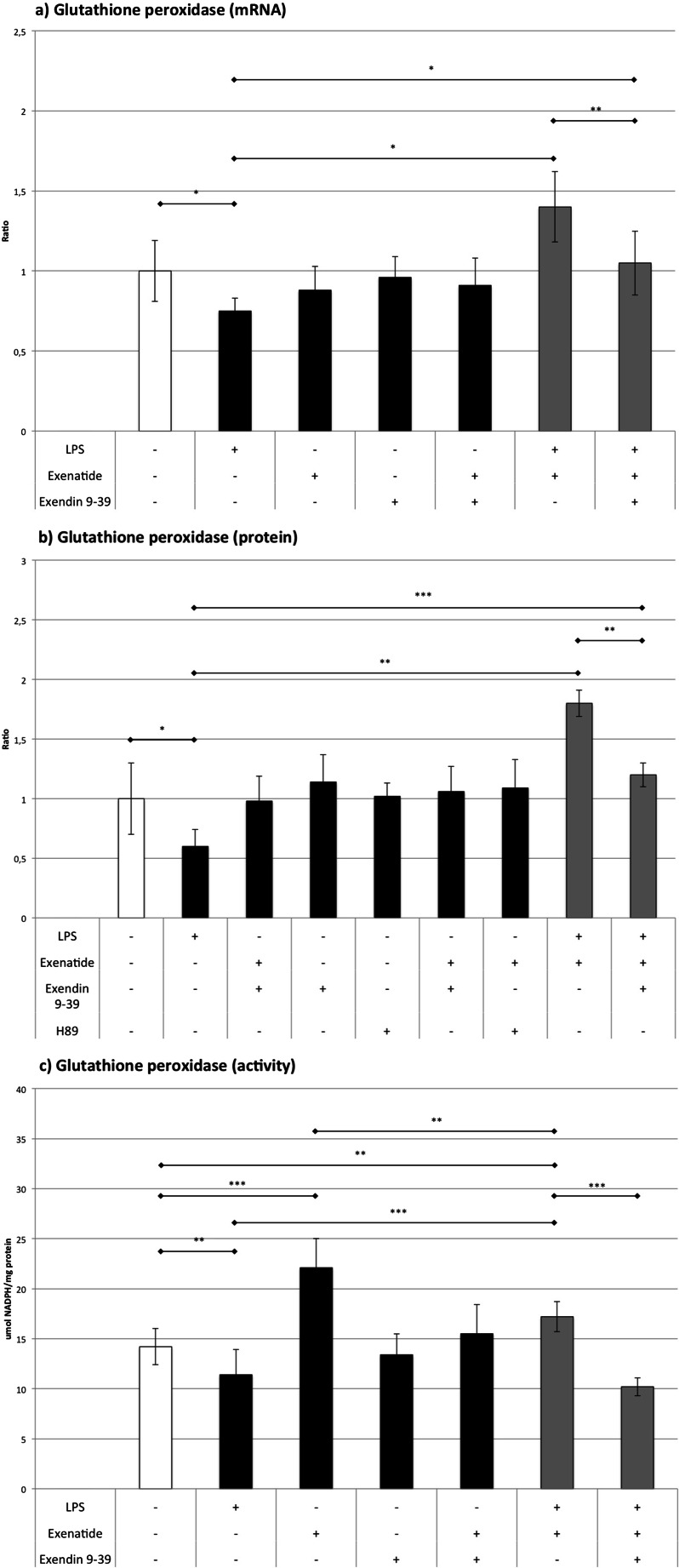
The influence of LPS, exenatide, exendin 9-39, and H89 (for Western blot only) on the expression of glutathione peroxidase assessed by **a** RT-QPCR and **b** Western blot analysis and **c** on the activity of the glutathione peroxidase. *p < 0.05, **p < 0.01, ***p < 0.001

LPS reduced mRNA and protein expression for glutathione peroxidase by 25 and 40 %, respectively. These findings were accompanied by a decrease in GSH-Px activity by 20 %. Exenatide did not influence the expression of GSH-Px mRNA and protein, but it elevated the GSH-Px activity by 61 %. Cells treated with LPS plus exenatide increased the expression of GSH-Px mRNA by 42 % and protein by 79 %. In this setting, the activity of GSH-Px was higher than in control (by 21 %) and cells treated with LPS (by 33 %). On the other hand, it was lower than in cells treated with exenatide (by 22 %). GLP-1 antagonist reduced the impact of LPS plus exenatide on the expression of GSH-Px mRNA (by 25 %) and protein (by 34 %), but the expression levels remained higher than in cells treated with LPS (by 39 % for mRNA and 102 % for protein). Furthermore, exendin 9-39 reduced the activity of GSH-Px in macrophages treated with LPS plus exenatide (by 41 %). These findings showed that exenatide elevated the activity of GSH-Px without influencing the level of expression of the enzyme. LPS treatment was associated with a decrease in enzymatic activity and GSH-Px expression. Co-stimulation with LPS and exenatide was associated with an increase in enzymatic activity connected with a rise in mRNA and protein expression, which supports the idea of an improvement of antioxidative potential of macrophages followed by exenatide.

### CAT (Fig. 6)

**Fig. 6:**
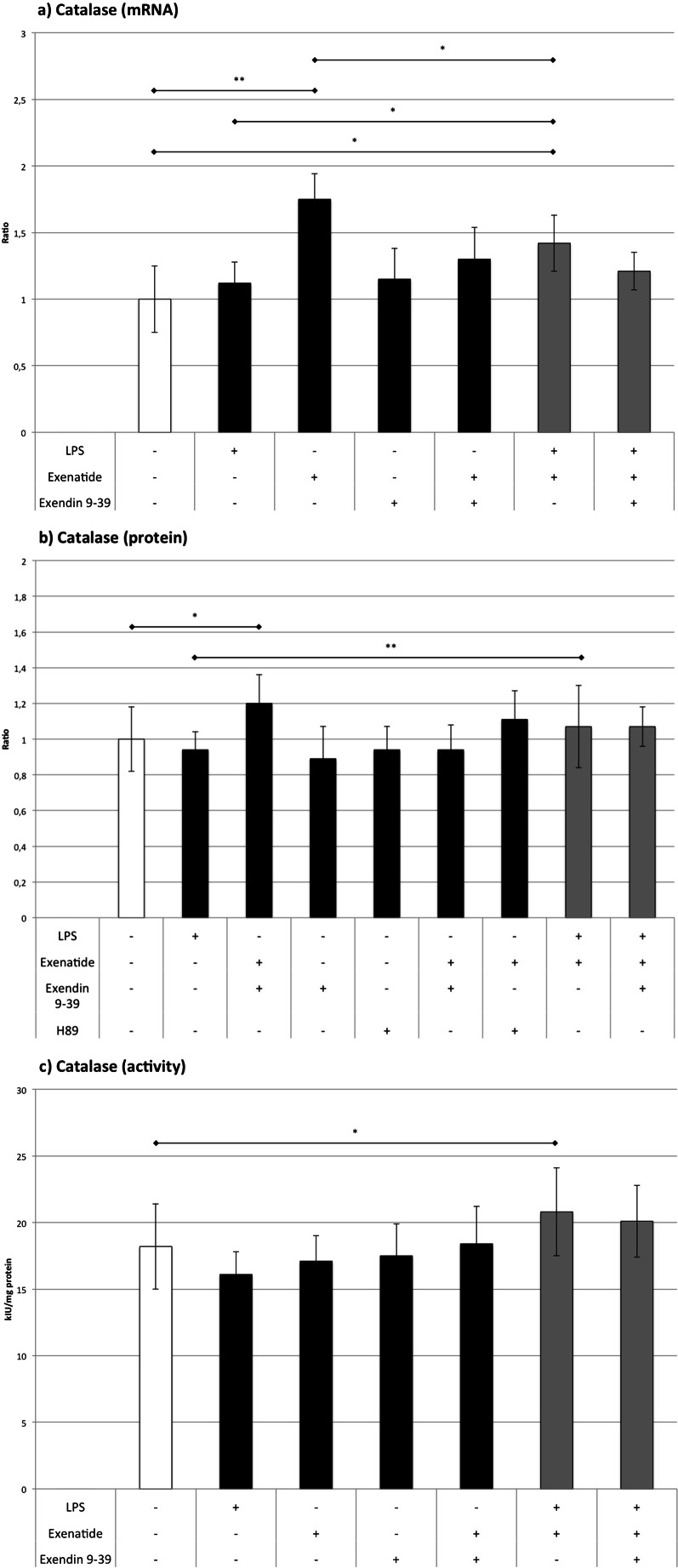
The influence of LPS, exenatide, exendin 9-39, and H89 (for Western blot only) on the expression of catalase assessed by **a** RT-QPCR and **b** Western blot analysis and **c** on the activity of the catalase. *p < 0.05, **p < 0.01, ***p < 0.001

Exenatide considerably elevated CAT mRNA and protein level by 75 and 22 %, but it did not change the activity of the enzyme. LPS did not affect the expression and activity of CAT. Compared to control, an increase in mRNA level was observed in cultures with LPS plus exenatide (by 44 %); however, this rise was smaller than that observed in cells only treated with exenatide (by 18 %). In the group treated with LPS plus exenatide, a modest elevation in CAT activity was observed (by 14 %) despite the lack of an influence on protein expression. Exendin 9-39 did not affect the enzymatic activity of CAT compared to LPS and exenatide. Exendin 9-39 did not reverse the exenatide-induced increase of enzymatic activity of CAT in the presence of LPS suggesting GLP-1 receptor independent effects (Sathananthan et al. 2013). These results are somewhat puzzling, but overall, it seems reasonable to assume that the influence of exenatide on catalase activity is at best modest and shows increasing activity with the simultaneous inflammatory stimulus.

### Experiments with H89 (Fig. 7)

**Fig. 7:**
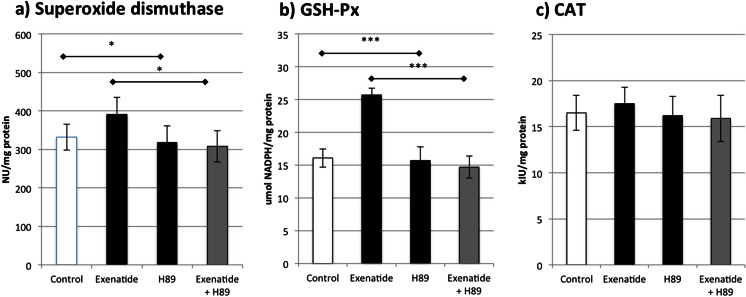
The influence of protein kinase A inhibitor (H89) on the activities of **a** superoxide dismutase, **b** glutathione peroxidase, and **c** catalase in monocytes/macrophages treated with exenatide. *p < 0.05, **p < 0.01, ***p < 0.001

According to the abovementioned experiments and the fact that most of the effects were blocked or tapered by exendin 9-39, we estimated the influence of H89 on the downstream protein kinases—predominantly PKA. Our results showed that H89 prompted complete inhibition of the influence of exenatide on SOD and GSH-Px activity, which resulted in levels comparable to those reported in control samples. On the other hand, we did not observe any effect of H89 on CAT activity. However, it should be noted that exenatide also did not affect the level of CAT activity.

## Discussion

Our study was conceived to estimate the oxidative and antioxidative potential of exenatide—a novel antidiabetic drug—on cultured human monocytes/macrophages. In our comprehensive approach, we estimated the influence of exenatide on untreated macrophages and those treated with LPS. The exposure to LPS was considered as conditions resembling inflammation—similarly to that observed in diabetes and atherosclerosis. Furthermore, the use of exendin 9-39 (GLP-1 antagonist) and H89 (protein kinase A inhibitor) gave additional insight into the regulation of redox status.

We showed that exenatide improved the antioxidative potential of cultured human monocytes/macrophages. The majority of effects were seen in cells pretreated with LPS. We observed a decrease in ROS and MDA level, which was accompanied by the reduced expression of NADPH oxidase. Additionally, we noted a significant rise in the expression and activities of SOD and GSH-Px, but the CAT expression and activity remained unaffected. The observed findings were reversible by exendin 9-39 and H89.

Macrophages are key cells in the progression of atherosclerosis, since their participation in the pathogenesis of diabetes and its complications have been strongly advocated for several years. These cells are responsible for a substantial portion of oxidative stress in living organisms, which is necessary to tackle the infections and parasitic infestations. However, nowadays, it is believed that the increased generation of ROS by macrophages results in the deterioration of beta pancreatic cells, which is described by the diminished secretion of insulin and increased insulin resistance (Maechler et al. 1999). In our experimental model, we noted that macrophages treated with LPS generated high levels of ROS and MDA. Conversely, the addition of exenatide resulted in a significant decrease in both of the abovementioned markers of oxidative stress. Similar results were seen in animal macrophages, human umbilical vein endothelial cells (HUVEC), and adipose-derived stem cells (Liu et al. 2014). The current experiment showed that the ROS inhibition by exenatide was not sufficient to restore the levels comparable to the control, untreated cells. The reason for this observation may be the fact that LPS is a very strong inflammatory stimulus that may not be completely blocked by other, unspecific toward toll-like receptor 4 (TLR4), mechanisms (Biswas and Mantovani 2012). Others have reported that exenatide effectively reduced ROS level in the in vivo treatment of human diabetic subjects (Chaudhuri et al. 2012). The potential mechanisms behind this phenomenon include the influence on the synthesis of ROS or on the antioxidative potential of macrophages.

NADPH oxidase level is one of the main sources of ROS (Foronjy et al. 2006), and its activity was shown to be elevated in chronic inflammatory state, which was reproduced in our experiments with LPS (Morgan et al. 2007). Additionally, we observed that exenatide significantly reduced the expression of NADPH oxidase p22 protein in LPS-treated macrophages. Similar findings were noted in renal tubular cells from streptozotocin-induced diabetic rats after liraglutide (another GLP-1 agonist) (Hendarto et al. 2012). Exendin 9-39 completely blocked the impact of exenatide on p22 expression and supports the concept that the GLP-1 receptor stimulation was completely responsible for the results. Therefore, we concluded that exenatide reduces the ROS level by reducing the expression of NADPH oxidase.

During the next step of our study, we wanted to assess whether the effects on ROS are also connected with an influence on antioxidative enzymes. In order to explore the impact of exenatide on antioxidative properties on macrophages, we measured the expression of SOD, GSH-Px, and CAT—three enzymes that are essential in resolving oxidative damage. LPS-treated macrophages showed elevated expression of SOD, which may reflect a compensatory mechanism, preventing cellular death resulting from uncontrolled oxidative stress. On the other hand, GSH-Px expression was reduced, and CAT expression remained unaffected. These findings, together with the influence on NADPH oxidase, may translate into increased oxidative stress associated with inflammatory stimuli. Exenatide significantly elevated the expression of SOD, GSH-Px, and CAT in LPS-treated macrophages. Similar findings for SOD and GSH-Px were reported in H9c2 myocytes and rodent macrophages (Chang et al. 2014; Murata et al. 2002). SOD is considered an important defense mechanism in the protection against atherosclerosis and pulmonary emphysema, both of which are associated with increased oxidative stress (Foronjy et al. 2006; Fujimoto et al. 2010). Reports on the effects of exenatide on catalase expression are scarce. Other researchers showed the increased expression of CAT in H9c2 cells (Chang et al. 2013a, b). This discrepancy may result from different cell lines studied and different culture conditions or the fact that a substantial portion of antioxidative features in macrophages is connected with the SOD and GSH-Px pathways (Murata et al. 2002).

In the next step of our study, we checked whether changes in the expression of antioxidative enzymes were accompanied by alterations in their activity. Exenatide markedly elevated the SOD and only modestly CAT activity in LPS-treated macrophages; these findings were concurrent with the expression level of the abovementioned enzymes. Interestingly, GSH-Px activity was slightly lower than in cells treated with LPS alone, despite a significantly higher protein level in the presence of exenatide. This observation may stem from the simultaneous reduction in NADPH oxidase expression and succeeding ROS generation, which eliminates the need for high antioxidative reaction. In our previous work, we showed that another antidiabetic drug, metformin, also affected the antioxidative potential of human macrophages. Similarly to the current study, the SOD and CAT activity was elevated in LPS-pretreated cells, but metformin also increased the activity of GSH-Px (Buldak et al. 2014). We believe that the reason for that difference is based on the different mechanism of action of these two drugs. Metformin acts by activating AMP-dependent kinase, while GLP-1 receptor stimulation results in PKA activation.

Finally, we explored the effects of PKA inhibition on the activity of antioxidative enzymes using a pharmacological inhibitor—H89. In order to reduce confounders, we performed studies without LPS pretreatment, because TLR4 also increases the level of intracellular cAMP and PKA. We showed that the influence of exenatide on SOD and GSH-Px was at least partly PKA-dependent, but we did not show any significant change in CAT activity. These results showed that antioxidative potential induced by exenatide directly relies on the improved activity of SOD and GSH-Px. It is difficult to compare our results with those of other researchers due to few published reports on the activity of antioxidative potential and the use of kinase inhibitors in human macrophages. However, others reported that PKA inhibition reduced the expression of iNOS—a protein associated with inflammatory type of macrophages and a source of reactive nitrogen species (Chang et al. 2013a, b). Additionally, a different protein kinase inhibitor (PKI) was shown to suppress the antiinflammatory phenotype induced by exenatide in endothelial cells and macrophages (Arakawa et al. 2010).

Limitations of our experiments should also be kept in mind: (1) We have used supratherapeutic concentrations of exendin, which is often necessary in in vitro studies to compensate the impact of drug given in vivo. In diabetic subjects treated with subcutaneously administered exenatide, plasma concentration of the drug averages between 50 and 75 pM (Drucker et al. 2008), which is significantly lower than 10 mM used in our experiments. The reason for that is the relatively short time of the exposure of cells to study drug in comparison to human subjects with diabetes that may take drug for several years. (2) The high dose of the reagent may in some cases induce unspecific actions that may require further investigation (e.g., experiments with catalase). (3) Finally, H89 is not selective to PKA but also inhibits other kinases (e.g., PKB/Akt, PKC μ), which might affect the findings. Other PKA inhibitors show more specific action toward PKA but may still display some unwanted effects. In order to pinpoint the involvement of protein kinase A, further experiments with other PKA inhibitors or preferably gene-silencing techniques are necessary.

## Conclusions

Our study showed that exenatide, a GLP-1 receptor agonist, significantly affect redox state in cultured monocytes/macrophages. These findings include a reduction in oxidative stress described by reduced ROS and MDA level and NADPH oxidase expression, which is accompanied by an improvement in antioxidative potential, being predominantly a consequence of increased expression and the activities of SOD and GSH-Px.
